# Zooplankton feeding behavioral signatures in the morphology of macroscale prey spatial distribution

**DOI:** 10.1371/journal.pcbi.1014411

**Published:** 2026-07-06

**Authors:** Eduardo H. Colombo, Corina E. Tarnita, Juan A. Bonachela

**Affiliations:** 1 Department of Ecology & Evolutionary Biology, Princeton University, Princeton, New Jersey, United States of America; 2 Department of Ecology, Evolution, and Natural Resources, Rutgers University, New Brunswick, New Jersey, United States of America; University of Maryland, UNITED STATES OF AMERICA

## Abstract

The problem of pattern and scale remains central in ecology, bridging fundamental and applied questions. Marine microbial communities are a case in point. For instance, to understand the role of zooplankton in oceanic biogeochemistry, their response to changes in environmental conditions, and the implications for ecosystem services (e.g., fisheries), it is critical to understand zooplankton trophic interactions and how they change in a rapidly changing climate. This understanding, however, remains elusive because, unlike for phytoplankton, for which remote sensing of macroscale patterns can provide insight into their microscale dynamics and community composition, obtaining this information for zooplankton largely rests on quantifying the difficult-to-monitor microscale interactions among millions of individuals with different behaviors, and between individuals and their environment. Here, we investigate whether it is possible to obtain indirect information on zooplankton from the macroscale spatial distribution of their prey. To tackle this “problem of scale,” we develop a rigorous coarse-graining methodology that connects individual-level properties with macroscale spatial patterns. We demonstrate that the shape of the prey spatial distribution can encode information about zooplankton feeding behavior and community dynamics. Specifically, we predict a change in dominant feeding behavior—from non-motile to motile feeding—as one moves from areas of high to areas of low prey density. These computational results are validated by our analysis of satellite images of oceanic blooms around the globe, which suggests novel opportunities for remote sensing approaches: the potential tracking of consumer behavioral signatures in the large-scale patterns of the resource. Importantly, the scaling-up methodology developed here to check for those signatures is general, and can be used to link scales rigorously and systematically in any system in which the complexity of individual dynamics makes connecting scales intractable.

## Author summary

Understanding how scales are connected is a fundamental problem in ecology. For plankton communities, this entails understanding how the complex microscale interactions among millions of individuals and with their environment ultimately result in exuberant kilometer-scale phytoplankton patterns (blooms), regularly monitored using remote sensing. Here, by successfully tackling this theoretical problem we unlock a novel use for remote sensing approaches. Applying a coarse-graining method upon an individual-level model for phytoplankton-zooplankton communities, we show that the shape of phytoplankton patterns can encode information about the feeding behavior of zooplankton. Our analysis of real-world bloom data suggests that similar zooplankton behavioral signatures can be indeed identified using phytoplankton remote-sensing images. This information can thus help identify indirectly the behavioral composition of the zooplankton community which, in addition to the dynamics of phytoplankton, influences the composition of the fish community and related ecosystem services.

## 1 Introduction

Marine ecosystems stand on the shoulders of microbial organisms. Phytoplankton—microbial primary producers—have a protagonist role as they recycle atmospheric carbon dioxide to generate more than half the oxygen produced on Earth every day, sequester carbon to the deep ocean in the form of sinking marine snow, and introduce carbon and other essential nutrients (e.g., nitrogen, phosphorus) into the marine food web [1–3]. Zooplankton, predators of phytoplankton and other prey (e.g., bacteria, other zooplankton) and preferential resource for fish, create a link that connects the lower to the higher trophic levels of the marine food web, as well as to the deep sea via excretion of dissolved and particulate organic matter [4,5]. Similarly to their prey, zooplankton are part of diverse communities with different functional roles, and thus have different contributions to ecosystem dynamics. In particular, the implementation of these dynamics (i.e., zooplankton grazing) has been identified as a major source of uncertainty for models aiming to forecast marine carbon cycling [6]. Understanding and predicting oceanic biogeochemistry and other key marine ecosystem services therefore requires understanding and predicting what zooplankton grazing behavior dominates at focal locations.

**Fig 1:**
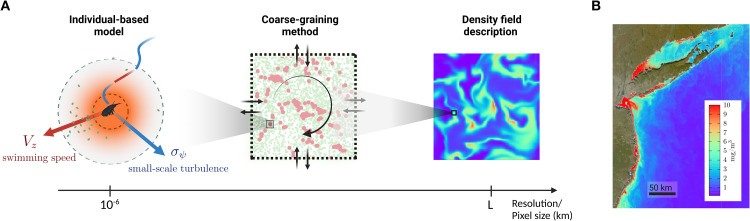
From the micro- to macroscale. **A)** Using our individual-based model as a starting point (left panel), we performed a coarse-graining method (middle panel) to obtain the interaction and transport terms for a density-field description (right panel). Our methodology provides a connection across scales of observation that facilitates understanding the large-scale consequences of the individual-level features such as the swimming speed of the focal zooplankton species, $V_{z}$, and the typical speed stemming from small-scale turbulence, $\sigma_{\psi }$ (see *Materials and methods*). **B)** Chlorophyll concentration (*Chl-a*) signal captured by HawkEye [7] close to the New Jersey - New York coastline (United States) on November 9, 2021.

Using chlorophyll as a proxy, remote sensing can determine the presence and even composition of large phytoplankton communities, revealing mesmerizing kilometer-scale phytoplankton spatial patterns (see Fig 1) with impressive spatial and temporal resolution [7,8]. Remote sensing, however, cannot in general directly provide information about other key players in the microbial food web (see [9] for a rare exception). Standard approaches to obtaining information about zooplankton and other important actors of oceanic carbon flow such as viruses involve *in-situ* observations, a much greater effort compared to remote sensing as it entails ship expeditions, large interdisciplinary collaborations, and substantial financial resources per case studied (e.g., [10,11]). Despite the large amount of data they can produce, these available methods can only offer limited information about species interactions or zooplankton feeding behavior [12–14]. Measuring and characterizing feeding behavior *in situ*, for example, is challenging without affecting (and thus biasing) observations [15]. Behavior, however, influences feeding rates, therefore affecting important ecological outcomes such as the flux of carbon towards the deep ocean and the structure of the microbial marine food web [16,17]. Thus, these limitations ultimately handicap the understanding of, and ability to predict, oceanic biogeochemical processes and how they change across space and time [13,18,19].

One way to address these limitations is to search for complementary indirect information in remote-sensing data (see examples of the use of models to infer zooplankton biomass [20–22]). Extracting information on zooplankton dynamics and feeding behavior in a non-invasive way using satellite images of, for example, macroscopic (kilometer-scale) patterns of phytoplankton or other prey would be an important next step to further our understanding of their role in those patterns. This is, however, a non-trivial and potentially infeasible endeavor due to the complexity of the links between the observed large-scale patterns and the individual-level interactions that influence them [23].

If this ‘problem of scale’ is generally difficult to solve for any given biological system, it is especially challenging in the case of zooplankton because of their feeding behavior. Although, for decades, macroscopic and even global biogeochemical models used the simplifying assumption that zooplankton are passive relative to oceanic turbulence [24,25], advances in high-resolution computer simulations [26] and lab-scale experiments [16,27,28] have stressed that zooplankton do not just ‘go with the flow’ [29]: a wide variety of zooplankton can sense the surrounding environment and use this information to decide where to swim [30]. As a result, a tug-of-war emerges between this impressive behavioral capability and turbulence, influencing where and when prey are eaten. At the microscale, this tug-of-war could lead to the formation of zooplankton patches [27,29,31] that potentially also affect the spatial distribution of, e.g., dissolved organic matter (resulting from zooplankton excretion) or particulate organic matter (from zooplankton faecal pellets or sloppy feeding), all of which could influence and contribute to the emergent large-scale patterns and could have rippling effects across the rest of the marine food web [17]. However, the mechanistic underpinnings of these patterns—i.e., whether, how, and what individual-level behaviors are responsible for shaping the patterns at different scales—are still unknown. This is in large part due to the technical intractability of connecting, step-by-step, the individual-level zooplankton behavior (well-known thanks to controlled experiments, e.g., [30]) with the associated microscale formations and, ultimately, with the resulting macroscale prey patterns, a connection that involves a broad spatial range (from millimeters to kilometers) and massive numbers of individuals to be tracked.

Despite this missing connection, existing theories have been able to reproduce qualitatively some features of large-scale oceanic plankton patterns by resorting to generic arguments or heuristic approximations that did not require explicitly linking individual and macroscopic levels [24,25,32–34]. For example, the fact that zooplankton and phytoplankton form distinct patterns has been captured by simple models with local facilitation and long-range competition among planktonic populations (e.g., [24]). Similarly, other studies have explored the role of zooplankton in the carbon cycle (e.g., assessed consumption rates) by using phenomenological population-level expressions that depend on individual traits [20,30,35,36]. In all previous theories, however, the lack of an explicit mechanistic connection between individual-, micro-, and macroscale properties leaves a causal gap that prevents the utilization of macroscale patterns to infer key aspects of the microbial community, such as the zooplankton feeding behavior that dominates. This causal gap also precludes theories able to predict potential shifts in the community over time, especially as anthropogenic effects promote substantial changes in ocean properties (e.g., toxicity [37], increased temperature, lowered viscosity, stratification [38] and turbidity [39,40]) that can result in behavioral changes at the individual level.

Here, we attempt to close this causal gap by introducing a novel computational coarse-graining method that allowed us to scale up an individual-based model to a macroscale density-field description (see Fig 1A). We show that zooplankton feeding behavior leaves fingerprints in prey patterns at multiple scales. At the microscale, the individual-level model reveals that zooplankton active search for resources (active behavior, i.e., swimming towards perceived prey) induces an expected increase in spatial correlation between prey and zooplankton but also an unexpected correlation among zooplankton, which ultimately form clumps. At large scales, our results show that the shape of the prey patterns can reflect the underlying feeding behavior, and thus that remote sensing can indeed be used to infer behavior: passive (i.e., non-swimming) zooplankton lead to smooth prey patterns while active zooplankton produce sharp pattern edges. We further extended our theoretical model to allow for the two types of behavior to explicitly compete for prey, which uncovered a change of dominance from passive to active feeding as the density of prey declines. In light of these predictions, we analyzed a collection of satellite images of phytoplankton blooms from different locations and seasons in search for zooplankton behavioral signatures. Our analysis suggests a change of dominance within blooms, from passive to active zooplankton feeding, as the density of phytoplankton declines from the center to the boundary of the pattern. This conclusion is confirmed by an extension of our theoretical model in which the two types of behavior explicitly compete for phytoplankton. Thus, by enabling the identification of spatial fingerprints of feeding in remote sensing products, our methodology translates macroscale phytoplankton features into meaningful information about the underlying individual-level interactions between zooplankton and phytoplankton. Such information could be used to, for example, identify the dominant zooplankton species (or, at least, the dominant behavior) across time and environmental gradients, and monitor shifts that might occur under climate change and that would ultimately influence the dynamics of the microbial community [15,41] and the rest of the marine food web. More generically, beyond marine systems, our framework can be used to check whether consumer (e.g., predator) behavioral signatures exist in the spatial pattern of the resource by just applying our coarsening method to individual-level data from the particular case under study. Here, we focus on zooplankton feeding, and thus henceforth we use zooplankton and predator interchangeably.

## 2 Results and discussion

We developed a general modeling framework that rigorously connects microscale dynamics to emergent macroscale patterns. The description of the macroscale arises from a coarse-graining method that considers the individual-based dynamics occurring in square domains (pixels) of size *L* that are stitched together, while keeping pixel size below the typical Kolmogorov scale of the ocean [42]. For scales below the pixel size, the individual-level dynamics are described by an individual-based model (IBM) where oceanic turbulence is effectively accounted for by considering that the velocity of both zooplankton and prey individuals is random with standard deviation set by parameter $\sigma_{\psi }$, a proxy for turbulence intensity at these scales. At the pixel scale and beyond, the coarse-graining method uses the IBM to derive transport, demographic, and interaction rates that, together, define partial differential equations for the predator-prey dynamics. We then coupled these emergent partial differential equations with a point-vortex model that accounts for large-scale turbulent structures (with intensity ψ, see *Materials and methods* and Fig E in S1 *Supplementary materials*). Below, we discuss first the microscale dynamics and subsequently the coarse-graining procedure and resulting macroscale patterns.

### 2.1 Microscale dynamics

The IBM accounts for the main processes generally observed across zooplankton and prey types (individual birth and death, zooplankton feeding behavior) and an external flow that aims to mimic the effects of turbulence. Feeding determines prey death rate and zooplankton reproduction rate, and is influenced by the interplay between physics (i.e., flow) and ecology (i.e., predator swimming behavior). At the scale of the IBM, the turbulence flow is represented by a random velocity field that moves individuals; this flow tends to break correlations between prey and zooplankton. In the ocean, zooplankton can perceive visual and physico-chemical cues (e.g., hydrodynamic disturbances and chemical trails), to which they respond in different ways [30]. In our model, we generically captured feeding behavior by defining a perceptual range $R_{p}$ and two types of responses to cues within that range: active and passive. Active zooplankton individuals sense prey individuals within the perception range, swim towards them at a speed $V_{z}>0$ (if speed is strong enough for the predator to overcome the flow) and, with some probability [43] and in a negligible time [44], catch and ingest the target when the prey is within a catching range $R_{c}<R_{p}$. Passive zooplankton individuals, on the other hand, do not swim (i.e., $V_{z}=0$) even if they might sense prey, but they ingest prey if the flow brings both prey and predator within catching range. Active predators in our model would thus resemble cruise feeders that intentionally swim towards prey, while passive predators would encompass zooplankton feeding modes that rely on currents to bring prey within catching range (e.g., ambush feeders) [30]. Finally, the frequent change in orientation and different perception modes observed for zooplankton allow us to assume, for both our active and passive grazers, a circular perception range (instead of a more realistic but technically challenging forward-facing wedge [45]). For further details, see IBM description in Fig 1A, *Materials and methods*, and S1 *Supplementary materials*, and parameter values in Table A (S1 *Supplementary materials*).

Although overall zooplankton behavior depends on various parameters (see above), here we focused on the interplay between flow and swimming behavior. To this end, we varied the small-scale turbulence intensity, $\sigma_{\psi }$, and the swimming speed $V_{z}$. Note that, although swimming entails a metabolic cost, we did not include it in this version of the model for the sake of simplicity. We did so, however, in an extension of the model specifically devised to contrast costs and benefits of feeding behavior (see adaptive-feeding model in *Materials and methods*).

#### 2.1.1 Emergent timescale separation.

Transport and demographic processes can both introduce spatial correlations and thus play a role in the emergent spatial pattern: the former, via physical spatial rearrangements of individuals; the latter, via changes in species densities (i.e., due to mortality/feeding, which decreases locally the number of individuals, or due to replication, which increases it). The model revealed, however, that transport and demography lead to correlations at different timescales. Given that typical speeds for flow and our generic zooplankton were on the order of ~1 cm/s and that the predator perceptual range is ~1 cm (see *Materials and methods*), transport-induced spatial features emerged in our simulations in just a few seconds. This timescale was much shorter (approximately 1/10) than the typical time interval between demographic events in the model. As shown in Fig F, such a timescale separation occurs for a wide range of prey densities. Importantly, this emergent timescale separation between the short-term relocation of individuals and demographic events allowed us to study these processes independently, as explained in the next two sections.

#### 2.1.2 On the short timescale, the movement of active zooplankton induces microscale patchiness.

**Fig 2:**
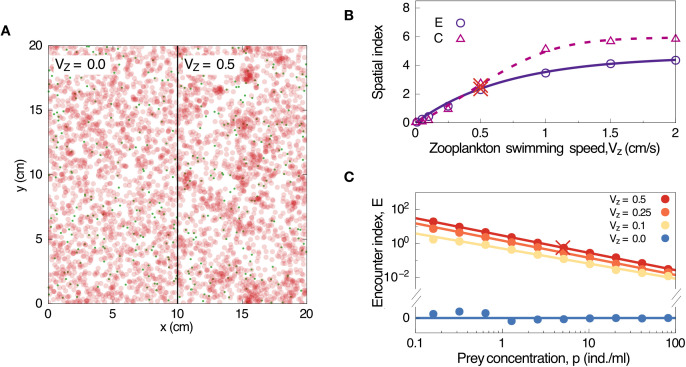
Microscale patchiness emerging from the individual-level model. **A)** Spatial distribution for prey (green dots) and predators (80%-transparent red dots) for densities (*p*,*z*) = (1.28,5); note that this example was chosen to illustrate the emergence of patterns, which are more pronounced at low prey densities. The left half of the picture shows the passive zooplankton case ($V_{z}=0.0$ cm/s), and the right half the active zooplankton case ($V_{z}=0.5$ cm/s), for easy visual comparison. **B)** The patterns from the scenario in panel A) are characterized by the encounter (*E*) and clumping (*C*) indices, shown here also for other zooplankton swimming speeds, $V_{z}$. **C)** Encounter index as a function of prey density for different swimming speeds $V_{z}$, showing that the benefits of active grazing with respect to the random case increase as prey density decreases. In both B) and **C)**, dots represent the mean across replicates of values reached in the stationary regime, with crosses corresponding to the $V_{z}=0.5$ case depicted in panel **A)**. Solid lines are given by Eq 1. All cases were obtained for moderate turbulence ($\sigma_{\psi }=0.25$ cm/s).

To understand the role of a given swimming speed $V_{z}$ in the formation of microscale patchiness we focused on the short timescales, where transport occurs in the absence of demographic processes. Thus, we assumed a constant density of prey and zooplankton (*p* and *z*, respectively), initially randomly distributed across space, and we explored a range of values for $V_{z}$, leaving the other behavioral traits (e.g., perception range) constant. Fig 2A illustrates the contrast between the typical patterns that emerged, after a short transient, for passive ($V_{z}=0$ cm/s) and active ($V_{z}>0$) zooplankton for low values of prey density. For the passive case, the turbulence-controlled transport maintained a random spatial distribution for both species. For active zooplankton, however, the directional movement towards prey increased the correlation between the location of zooplankton (red dots) and prey (green dots) and, unexpectedly, also among zooplankton individuals (for details, see species pair correlation function in Fig G). The latter correlation resulted from predators that were sufficiently close to each other and therefore partially shared the same cues, which led them to move in the same direction.

To quantify the formation of microscale patchiness, we defined two spatial indices: the encounter index, *E*(*t*), related to prey-zooplankton encounters, and the clumping index, *C*(*t*), indicative of zooplankton aggregation (see *Materials and methods*). We defined both indices to be zero in the well-mixed (random) scenario and exhibit positive values in the presence of microscale patchiness (Eq 6). Figs HA and HB in S1 *Supplementary materials* illustrate how these spatial indices started at a zero value and, after a short initial transient (5−10 s), reached (quasi-)stationary values, *E* and *C*, which are our values of interest henceforth.

These stationary values, which characterize the microscale patchiness for the given densities (*p*,*z*), depended on predator swimming speed (Fig 2B) and turbulence (Figs HC to HF). For high values of $\sigma_{\psi }$, at any fixed $V_{z}$, turbulence generated displacements that were large relative to the zooplankton catching range, $R_{c}$, which resulted in random mixing (E~0, C~0). At moderate values of $\sigma_{\psi }$, *E* and *C* increased with $V_{z}$ and eventually reached a plateau when swimming offset turbulence, as predators could reach and stay close to selected prey targets (Fig 2B). At low turbulence, i.e., displacements smaller than the catching range, *E* and *C* increased with the swimming speed but showed a maximum at a finite speed $V_{z}^{⋆}$ (Figs HC and HD). For the remainder of our results, we focus on realistic speeds, which are below $V_{z}^{⋆}$ (see *Materials and methods*; we also explored what underlies this interesting peak via a simple model, see S1 *Supplementary materials* for details). All together, these results show that the tug-of-war between mixing (caused by the turbulent flow) and aggregation (caused by feeding behavior) determines species encounters (Figs HE and HF) and, consequently, potentially influences population-level interactions.

#### 2.1.3 Microscale patchiness is density-dependent.

Because they result from the relocation of individuals, the patterns emerging from the short-timescale processes above influence species interactions and the dynamics of species densities occurring at the longer timescale. In turn, demographic changes locally add and remove individuals and therefore affect short-term spatial correlations, how many individuals are transported, and the directions that active zooplankton will move into on the shorter timescale. This feedback loop shapes the emergent microscale patterns and how they change over time.

To investigate whether and how prey and predator demography influences the dynamics of microscale patchiness, we considered a given turbulence-speed scenario, i.e., fixed $\left(\sigma_{\psi },V_{z}\right)$, and monitored (*p*(*t*),*z*(*t*)) over time. Due to *t*he *t*imescale separation, individuals quickly rearranged in space between demographic events, which allowed us to define a stationary encounter and clumping index for each density, i.e., *E* = *E*(*p*,*z*) and *C* = *C*(*p*,*z*). We found that, in fact, both indices were significantly correlated only with prey density and not with zooplankton density, i.e., *E* = *E*(*p*) and *C* = *C*(*p*) (Figs I).

Fig 2C shows the emergent correlation between the encounter index and prey density under different swimming speeds. High prey densities led to frequent encounters with zooplankton similarly to the random encounters expected in the well-mixed scenario (i.e., E→0 as p→∞); as prey density decreased, however, encounters became rare unless the predator actively swam towards prey, and therefore active feeding substantially increased encounters relative to the passive case. Active feeding also increased the fraction of time predators had prey within reach (see S1 *Supplementary materials*). Both the encounter and the clumping indices (Fig I) show that active feeding promotes aggregation at low prey densities, but does not provide any significant advantage at high densities.

From Fig 2C we infer that the encounter index scales as a power law with prey density and depends on both small-scale turbulence and zooplankton swimming speed, i.e.,:


$$\begin{array}{c} E≃\frac{\alpha (\sigma_{\psi },V_{z})}{(p/p_{0}{)}^{\beta (\sigma_{\psi },V_{z})}}\;, \end{array}$$ (1)

where *p*_0_ = 1 ind./cm^2^ is a (fixed) scaling factor (see Fig 2C) and the coefficients α and β (unitless), both of which depend on turbulence and swimming speed $\left(\sigma_{\psi },V_{z}\right)$, can be extracted by fitting Eq 1 to the simulation data in Fig 2C (see Fig J for the fitting procedure). The numerator, $\alpha (\sigma_{\psi },V_{z})$, increases with swimming speed but decreases with turbulence intensity, thus encapsulating the aforementioned tug-of-war between turbulence and feeding behavior (Fig JA). The exponent β sets the degree of nonlinearity of the density-dependent encounter rates in Eq 1. Overall, β could be approximated to β≃1.0 (Fig JB), with deviations towards smaller values occurring only when the community is well-mixed (high $\sigma_{\psi }$ and low $V_{z}$), a regime in which α is negligible and, thus, E→0. This nonlinearity and consequent saturation at low prey densities that emerges from zooplankton feeding behavior alone (Figs JC and JD) provides ecological support for the use of Type III-like functional responses in ecosystem models [46], which typically need to assume low-density saturation for zooplankton consumption to ensure the stability of simulations [47].

Because, instead of making heuristic arguments, we extracted the encounter rate from our simulation data, Eq 1 brings a substantial improvement over previous theoretical proposals [30]. Moreover, Eq 1 allowed us to identify how zooplankton encounters with prey are affected by the environment (i.e., turbulence and prey density) and feeding behavior (i.e., zooplankton swimming speed), key pieces of mechanistic information that allow us to derive next the population-level consequences of microscale patchiness.

### 2.2 Coarse-graining: From micro to macro

The high computational cost of the IBM makes it suitable only for small systems (on the order of decimeters) and, therefore, it cannot be used to address the kilometer-scale plankton patterns observed in the ocean. Thus, to study the macroscale consequences of the microscale patterns above and their underlying population-level interactions, we implemented a coarse-graining method.

The method utilizes the idea from Renormalization Theory [48,49] that only key components of the microscale dynamics remain relevant at coarser scales and thus are responsible for the resulting large-scale phenomena, as the effects of irrelevant components “average out” at such scales. In other words, a coarser description obtained by looking at the microscale dynamics at larger scales (by, for example, calculating densities at a larger spatial unit of observation) will ultimately only retain the key components of those dynamics. Thus, to obtain the density-field description, we first considered a large square system of linear size ℒ composed of smaller sections (pixels) of size *L* that were computationally suitable for the IBM. Each pixel was assigned a coordinate (*i*,*j*) within the larger lattice. $P_{i,j}$ and $Z_{i,j}$ denote respectively the number of prey and zooplankton individuals within pixel (*i*,*j*), while $p_{i,j}\equiv P_{i,j}(t)/L^{2}$ and $z_{i,j}\equiv Z_{i,j}(t)/L^{2}$ represent their corresponding densities. Thus, instead of tracking individuals within each (IBM-friendly) pixel, we calculated the corresponding pixel-specific densities.

Under this coarse description, we followed a computational approach that borrows concepts from probabilistic simulation methods [50–52] to understand the dynamics of such densities. Specifically, we tracked the changes in species density within each time interval, Δt, for different turbulence-velocity scenarios $\left(\sigma_{\psi },V_{z}\right)$. In addition to the IBM dynamics within each pixel, individuals can also move between adjacent pixels (either due to flow or behavior, following the IBM rules). Thus, we measured *p* and *z* at the focal and adjacent (top, bottom, left and right) pixels at the beginning of the time interval, with $\Delta p_{+}(t)$ and $\Delta p_{-}(t)$ representing increases and decreases in prey abundance within the time interval and $\Delta z_{+}(t)$ and $\Delta z_{-}(t)$ those for zooplankton. See *Materials and methods* for further details.

Because we tracked the fate of each individual in the whole system, we could keep track of the changes due to birth-death processes (B) and those due to transport (T), i.e., $\Delta p_{\pm }=[\Delta p_{\pm }^{B},\Delta p_{\pm }^{T}]$ and $\Delta z_{\pm }=[\Delta z_{\pm }^{B},\Delta z_{\pm }^{T}]$. We then defined the rate at which density changes due to the former as $F_{p}=\Delta p_{\pm }^{B}/(\Delta t)$ and $F_{z}=\Delta z_{\pm }^{B}/(\Delta t)$, and the latter as $\Gamma_{p}=\Delta p_{\pm }^{T}/(\Delta t)$ and $\Gamma_{z}=\Delta z_{\pm }^{T}/(\Delta t)$. Importantly, the timescale separation between spatial movement and demographic events further resulted in a Markovian property of the coarse representation such that the changes in the densities of a given pixel, $\left(\Delta p_{\pm },\Delta z_{\pm }\right)$, could be determined by its current densities and those of its adjacent pixels [53]. For pixels large enough for microscale patchiness to emerge (i.e., *L* > 10 cm; see Fig L), these changes in density obeyed Poisson statistics (Figs MA and MB) with well-defined size scaling laws (Figs MC and MD) and mean determined by species densities, traits, and environmental conditions (Figs ME to MH). Altogether, we obtained a density field description for prey and zooplankton at time *t* and at every location, $\vec{r}=(x,y)$, within the larger system, where x=i·L and y=j·L translate from pixel integer coordinates to real location within the ℒ system:


$$\begin{array}{c} \partial_{t}p(\vec{r},t|L,𝒬)=F_{p}(p,z|L,𝒬)+\Gamma_{p}(p|L,𝒬)\;,\\ \partial_{t}z(\vec{r},t|L,𝒬)=F_{z}(p,z|L,𝒬)+\Gamma_{z}(p,z|L,𝒬)\;, \end{array}$$ (2)

These dynamics depend on the IBM parameters $𝒬\equiv \{\sigma_{\psi },V_{Z},\ldots \}$ and pixel size *L*, the latter setting the spatial resolution of the description (see *Materials and methods*).

Macroscale spatial patterns are typically monitored at pixel resolutions for which the IBM is computationally intractable (e.g., for phytoplankton patterns in the oceans, resolutions range from 120m for the high-resolution satellite images from the SeaHawk Cube Sat mission [7] to 1km for the well-known MODIS database [8]. Therefore, in order to understand what our theory would predict when utilizing (or comparing with) available remote-sensing data, we needed to derive Eq 2 explicitly for large pixel sizes. To this end, we simulated the IBM for several sizes up to the upper computational limit (*L* = 100 cm) and used these simulation data to extrapolate the behavior to larger pixel sizes (see S1 *Supplementary materials*). This extrapolation was enabled by the well-behaved statistics described above (Figs MA and MB). Ultimately, for pixels of sizes comparable to the typical resolution of empirical data, this procedure yielded the following expressions:


$$\begin{array}{c} \partial_{t}p(\vec{r},t)=rp(1-p/K)-zg(p)-L^{-1}{\vec{v}}_{f}(\vec{r},t)\nabla p\;,\\ \partial_{t}z(\vec{r},t)=-mz+bzg(p)-L^{-1}{\vec{v}}_{f}(\vec{r},t)\nabla z\;. \end{array}$$ (3)

The first term of each equation represents the reproduction and mortality of prey and zooplankton, respectively; these logistic and linear forms, derived using data from our mechanistic IBM, match the terms typically assumed phenomenologically in population-level feeding models. The last term in each equation accounts for the advection (i.e., directional movement) of individuals due to the flow at the scale of the pixel, which moves around the planktonic predator and prey at the same velocity as the turbulent flow associated with eddies, ${\vec{v}}_{f}(\vec{r},t)$ (see *Materials and methods* Eq 8). An additional feeding-related directional movement also exists but becomes negligible at the kilometer scale when compared to the advection caused by the flow; its demographic effects, however, remain present for all *L*, leading to the second term in both equations, where the density-dependent function *g* encapsulates the microscale patterns induced by active feeding.

As explained in S1 *Supplementary materials*, further steps in our coarse-graining procedure showed that the per-capita zooplankton feeding function depends on the encounter index *E* and the catching range $R_{c}$; specifically, $g(p)=c\pi R_{c}^{2}p(1+E(p))$. As Fig J shows, this density-dependent term is different from the Holling type I or II functional responses [54] typically used to represent feeding in phenomenological models [55]. For instance, when *E* = 0 (i.e., well-mixed scenario and/or passive zooplankton), the ingestion rate vanishes as p→0, which allows the recovery of the local prey population. In contrast, when *E* > 0 (i.e., active zooplankton) feeding behavior compensates for low prey availability, which leads to high ingestion rates, prey population declines and, potentially, extinction.

Such different behaviors can have substantial consequences for the stability of the community dynamics, which can be illustrated by looking at the mean-field limit (L→∞) of Eq 3. When feeding is passive, we recapitulate the results of the Lotka-Volterra model (Fig NA). However, when feeding is active, trajectories in the (*p*,*z*) space do not match the typical expectations for this classic model (compare Fig NA to panels B and C). Specifically, for active feeding we found neither periodic oscillatory dynamics when the carrying capacity was assumed infinite nor inward spirals (i.e., damped oscillations that lead to stationary values) when the carrying capacity was assumed finite. Instead, the data-derived density-dependent interaction term created outward spirals (i.e., unstable oscillations) that can ultimately lead to the extinction of the community. See full linear stability analysis in Figs ND and NE.

In summary, passive feeding leads to qualitatively different population-level dynamics than active feeding, which might ultimately impact the spatial organization of the community at the macroscale. All these results set us up to tackle our original questions: whether and how macroscopic spatial data can be used to identify behavior across space and time in realistic scenarios. With this in mind, in the next section we adapt Eq 3 to study macroscale prey pattern formation.

### 2.3 Macroscale dynamics

Oceanic blooms are a paradigmatic example of macroscopic phytoplankton patterns. Phytoplankton blooms refer to the massive cell reproduction event that occurs seasonally as days become longer but temperatures are not yet high enough to significantly stratify the water column and therefore nutrients are plentiful in the photic zone. This demographic explosion leads to bursts of biochemical transformations that punctually alter biogeochemical cycles [56]. Blooms therefore co-occur with strong spatiotemporal heterogeneity of abiotic factors at various scales (e.g., nutrient availability and/or large-scale turbulence [57]) which, in combination with dispersal by turbulence, generate complex large-scale spatial patterns of phytoplankton that can be monitored through the remote sensing of chlorophyll.

#### 2.3.1 Grazer behavioral signatures in phytoplankton blooms.

**Fig 3:**
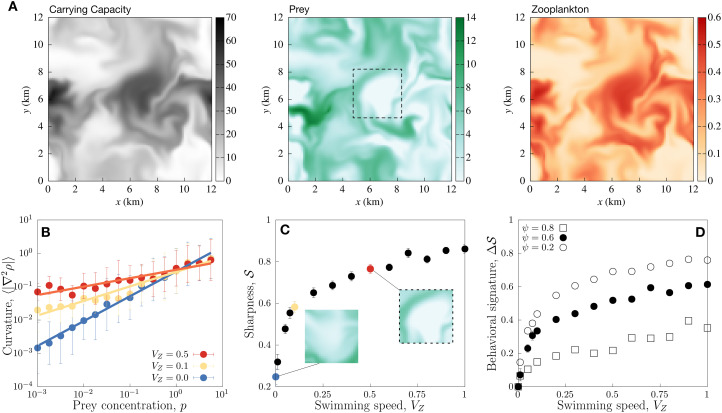
Zooplankton behavioral signatures in macroscale patterns of prey. **A)** Spatial patterns for carrying capacity, prey, and zooplankton (respectively, from left to right) in our simulated upwelling event. Color indicates densities in ind./ml. **B)** Curvature-density relationship for different swimming speeds, $V_{z}$. **C)** Pattern sharpness (i.e., 1 - slope of curvature) as a function of $V_{z}$, with colored dots referring to the cases in panel **B)**. Insets highlight typical patterns for $V_{z}=0.0$ cm/s and $V_{z}=0.5$ cm/s (the latter corresponding to the dashed square in the prey pattern from panel **A)**). **D)** Behavioral signature, defined as difference in sharpness relative to the passive case, $\Delta 𝒮\equiv 𝒮(V_{z})-𝒮(0)$, for different turbulence levels, ψ. For these simulations, the total domain size was ℒ=12 km (100 cells of size *L* = 120 m) and a horizontal carrying capacity band, $\bar{K}$, of length 12 km, width $\sigma_{K}=840$ m, and amplitude *K*_0_ = 100 was placed around the middle of the vertical axis. We adjusted eddies to introduce a mean flow speed ≃1 km/day (ψ=0.6 day^−1^) and fixed $\sigma_{\psi }=0.25$ cm/s to keep the diffusion and advective timescale comparable to experimental values [34,42]. The snapshots used for these figures were taken close to the peak of prey density during the upwelling event (approx. *t* = 12 days, as seen in Fig PA). We initialized the system using low, homogeneous prey and zooplankton densities (1 and 10^−2^ ind./cm^2^, respectively). See Table B in S1 *Supplementary materials* for parametrization.

To study whether zooplankton behavioral signatures can be identified in such a dynamic realistic scenario, we simulated upwelling events in the oceans, during which surges of nutrients lead to a wide range of densities for prey targeted by zooplankton. To this end, we modified Eq 3 in two ways. First, to account for the effects of large-scale eddies in the ocean, we added a point-vortex model (see *Materials and methods*) that set the net flow velocity for each pixel, ${\vec{v}}_{f}(\vec{r},t)$, (see Fig E) with the intensity parameter, ψ, controlling the angular speed of the eddies. Second, we introduced spatially explicit dynamics for the prey carrying capacity, $K(\vec{r},t)$, to model the heterogeneity of a growth-limiting nutrient that sets the maximum population density in the absence of predation. Specifically, the carrying capacity followed the equation:


$$\begin{array}{c} \partial_{t}K(\vec{r},t)=-\tau^{-1}(K-\bar{K})-L^{-1}{\vec{v}}_{f}\nabla K\;, \end{array}$$ (4)

where $\bar{K}(\vec{r})=K_{0}e^{-(y-ℒ/2{)}^{2}/(2\sigma_{K}^{2})}$ mimics a continuous input of nutrients occurring during the upwelling event that, if not depleted or perturbed by consumers, forms a horizontal band of width $\sigma_{K}$ in the center of a ℒ=100-km squared domain (Fig OA). The parameter τ represents the typical time for any initial condition for $K(\vec{r},t)$ to converge to the horizontal band $\bar{K}(\vec{r})$ in the absence of turbulence; we set τ=8 days, per [34]. For negligible eddy effects, the velocity of the flow is $v_{f}=0$ and thus $K\to \bar{K}$, but *K* will deviate from the imposed profile for moderate and strong eddies (Fig OB and first panel of Fig 3A). We initialized this dynamic carrying capacity as $K(r,0)=\bar{K}(\vec{r})$ with the aim of understanding how prey dynamics and turbulence disrupt such a marked initial pattern (i.e., the band); for robustness, we also explored other carrying capacity profiles (Figs OC and OD), which did not alter our conclusions.

With this setup, we integrated numerically our complete large-scale model following standard schemes (see S1 *Supplementary materials*) and studied how the spatio-temporal prey pattern depends on predator swimming speed, with the goal of discerning signatures of feeding behavior. In all cases, we initialized both zooplankton and prey populations using small densities. Regardless of the (fixed) $V_{z}$ value, the initially negligible top-down regulation allowed prey to grow rapidly to a peak of high density (Fig PA and prey panel in Fig 3A). The prey surge then facilitated the growth of predator density (zooplankton panel in Fig 3A), which consequently led to a decrease in prey density (Fig PA). Consistent with our mean-field findings, passive feeding led to stable coexistence of prey and predators, whereas active feeding ultimately led to the extinction of prey.

As this realistic and expected sequence of events unfolded, zooplankton dynamically shaped the emerging prey patterns. Importantly, the wide range of prey densities produced in space and time by these events offered an opportunity to investigate whether the density-dependent feeding effects described in the previous section were present in these macroscale spatial patterns. Based on our results in Fig 2C, we hypothesized that behavioral signatures emerge and should become more evident closer to the expanding edges, where prey density is lower. Specifically, we predicted that active feeding should produce a more noticeable contrast between the interior of the prey patterns and the borders (i.e., sharper boundaries) than passive feeding. Although visually that seemed to be the case (see, e.g., Fig PB), we also quantified the morphological differences caused by different swimming speeds by measuring the dependence between the curvature of the pattern and prey density [58]:


$$\begin{array}{c} 𝒞(p)=|\nabla^{2}p|\;. \end{array}$$ (5)

The function 𝒞(p) provides a proxy for the curvature of the prey density field *p* because, for a given location $\vec{r}$, $\left|\nabla^{2}p\right|$ quantifies density differences in any direction of the two-dimensional system. For smooth patterns, curvature vanishes as prey density asymptotically goes to zero. For sharp boundaries, in contrast, prey density abruptly drops to zero and, consequently, the curvature takes a non-zero value (see *Materials and methods* for further details and examples).

Using Eq 5, we measured curvature at any given time and every location in our simulated patterns, and extracted a relationship between curvature and prey density. We observed that this relationship approximated a power law throughout the duration of the upwelling event, although its slope varied with time (compare *t* = 2,12,14 in Fig PC). This power law emerged from the interaction with zooplankton and, at any given time, its slope reflected their feeding behavior (i.e., the magnitude of $V_{z}$; see Fig 3B and Fig PC). In particular, the slope captured clearly the difference between active and passive predators, which we traced to differences in swimming behavior rather than in overall increase in ingestion (passive zooplankton with a higher ingestion rate cannot induce similar signatures, see Fig PD). In other words, this finding confirmed our hypothesis that feeding induces behavioral signatures in prey macroscale patterns, and that these signatures can be inferred by measuring the curvature of boundaries or, more specifically, the difference in its slope. Thus, to quantify the sharpness 𝒮 of the boundary at a given time we used the slope of the curvature-density relationship: log⟨𝒞(p)⟩~(1−𝒮)logp, where ⟨𝒞(p)⟩ is the pattern curvature averaged over pixels with prey density *p*.

With the expression above, we could infer a sharpness for any given swimming speed, $𝒮=𝒮(V_{z})$ (see slopes in Fig 3B, and summary panel for 𝒮 as a function of $V_{z}$ and time in Fig PE). An ideal observation period encompasses the peak of prey density during the upwelling event because that window exhibits the widest range of densities that result from model dynamics. We indeed observed fingerprints of feeding behavior in sharpness (Fig 3C), as 𝒮 showed a very low value for simulations with passive zooplankton ($V_{z}=0$), and increased smoothly but steeply when we instead considered active zooplankton ($V_{z}>0$). A similar phenomenology was observed at other times across the duration of the upwelling event (Fig PE). Therefore, sharpness reflects behavioral signatures, and this abrupt change in value provides a way to discern between passive and active predators. The ability to notice these differences depended on turbulence: as one might expect, at high turbulence (large ψ) the strong eddies blurred behavioral signatures (Fig 3D).

#### 2.3.2 Analysis of real blooms.

**Fig 4:**
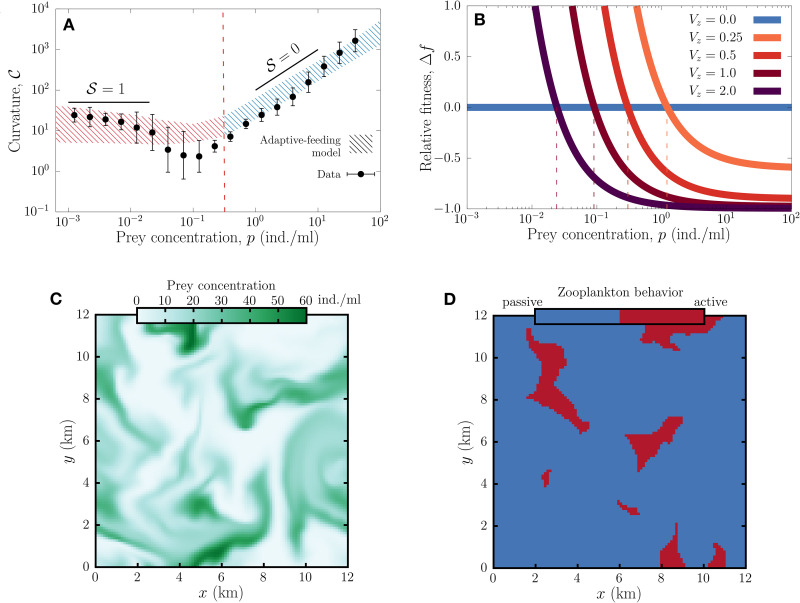
Spatial niche partitioning. **A)** Curvature-density relationship extracted from our adaptive-feeding model with $V_{z}=0.5$ cm/s (colored striped band) and from SeaHawk data (dots and error bars considering blooms across different locations and time of the year, see Table C in S1 *Supplementary materials*). The simulation estimates result from averaging over 50 snapshots (to account for the different sources of variability such as initial condition and eddy distribution) taken within a 4-day time-window right after the upwelling event; the vertical line indicates the density threshold identified in panel A for $V_{z}=0.5$, and colors (see colorbar in panel D) indicate the dominant behavior predicted by the adaptive-feeding model for each prey density. **B)** Relative difference, $\Delta f=(f_{1}-f_{0})/f_{0}$, between fitness for active ($V_{z}\neq 0$, with *f*_1_) and passive ($V_{z}=0.0$, with *f*_0_) zooplankton for different swimming speeds. Vertical dashed lines highlight, for each swimming speed, the prey density threshold below which being active is advantageous (i.e., value of *p* below which Δf>0). **C)** Prey density and D) zooplankton behavior patterns for the adaptive-feeding model with $V_{z}=0.5$ cm/s, for a snapshot close to the peak of the upwelling event. To match realistic concentrations, we set $K_{0}=10^{5}{\text{cm}}^{-2}$ and remaining parameters as in Fig 3A. For all panels, *q* = 4, $m_{1}=m_{0}=0.10$ day^−1^, $c_{0}=10\;ind^{-1}{\text{day}}^{-1}$.

Motivated by our findings above, we analyzed the sharpness of real blooms across the globe in search of behavioral signatures (see example in Fig 1B and snapshot locations in Fig SA). Specifically, we processed high-resolution images [7] and, assuming a reasonable conversion factor between chlorophyll-a and phytoplankton density [59,60], we obtained the corresponding curvature-density relationship (see *Materials and methods* for the protocol that allowed us to select snapshots around the peak of blooms). Whether we look at one or all locations, the data reveal a richer scenario than the one produced by our model for any given $V_{z}$. Differently from, e.g., Fig 3B, real-world blooms exhibit a curvature function with a density-dependent slope, which yields a density-dependent sharpness function 𝒮(p) that transitions abruptly from 𝒮≈0 to 𝒮≈1 as phytoplankton density decreases (black dots Fig 4A). Since our theoretical results indicate that active and passive behavior are associated with high and low sharpness, respectively, the real-bloom data suggest the existence of behavioral segregation, with passive grazers dominating at high densities and active grazers at low densities.

#### 2.3.3 Behavioral diversity can induce spatial niche partitioning.

To test our behavioral-segregation hypothesis, we extended our model—which, for simplicity, considered that all zooplankton had the same fixed feeding behavior, $V_{z}$—to allow for behavioral diversity. Zooplankton communities typically show diversity in feeding behavior [30], with some species even showing adaptive (i.e., context-dependent) feeding [61,62]. There are alternative approaches to making the simplest modeling extension that allows for behavioral diversity, and here we explored two of them for robustness: (i) we assumed two co-occurring species of zooplankton, one passive and one active (see S1 *Supplementary materials* and Fig R); alternatively (ii) we assumed a single species of zooplankton able to adapt its feeding behavior, i.e., change from passive ($V_{z}=0$) to active ($V_{z}>0$) feeding depending on metabolic costs and benefits [63].

Below, we focus on (ii) and modify Eq 3 such that the feeding function changes dynamically reflecting the differences between the metabolically costly but informed swimming associated with active feeding, and metabolically cheap “sit-and-wait” feeding associated with passive feeding that relies on random encounters (see Eqs 9 and 10 in *Materials and methods*). In this extended version of the model, at each point in space and time zooplankton show the feeding behavior that yields the higher ratio between ingestion and metabolic cost (i.e., higher fitness; see *Materials and methods*). As a result, passive feeding is beneficial at high prey densities while active feeding is beneficial at lower prey densities (Fig 4B). Because velocity is an essential component differentiating feeding behavior (and thus the feeding function), the emergent prey density threshold that determines whether active or passive feeding is advantageous is velocity-dependent (Fig 4B). Fig 4C shows a snapshot of the prey density obtained with the adaptive-feeding model at the peak of an upwelling event, while Fig 4D shows the behavior of the predator population in the same snapshot. Together, Fig 4B to 4D confirm that, when accounting for the main energetic costs of feeding even in this simplistic way, zooplankton switch behavior dynamically, with active behavior dominating at low prey densities and passive behavior dominating at high densities. Thus, these panels support the data-informed hypothesis of behavioral segregation, with active behavior dominating at low phytoplankton densities and passive behavior dominating at high densities.

Such behavioral segregation can indeed be inferred from the shape of prey patterns just by monitoring sharpness, 𝒮(p). As the model results in Fig 4A show, the sharpness of the pattern follows two distinct regimes that are in agreement with the data from real blooms. Specifically, both model and data indicate a sharpness transition from low (𝒮≈0) to high (𝒮≈1) values as prey density decreased (Fig 4A). Moreover, the model also closely predicted the phytoplankton threshold density associated with the transition from passive to active zooplankton behavior, a consequence of our ecologically reasonable minimal model and Chl-a conversion factor. Sensitivity analyses using different input profiles $\bar{K}$ (band versus hotspots) confirmed that this sharpness transition and its associated phytoplankton density threshold are robust across different upwelling scenarios (Fig QA). Varying the costs and benefits of active versus passive feeding produced different thresholds, but the overall qualitative profile of the curvature–density relationship remained unchanged (Fig QB).

The robustness of our findings was further reinforced by the similar qualitative results that we obtained under a scenario of behavioral diversity where we extended the original model to include an only-passive zooplankton species and an only-active species (scenario (i) above, see Fig R). The two species were able to coexist precisely because of an emergent spatial niche partitioning, whereby the passive species outcompeted the active one at high prey densities, but was outcompeted by the active one at low prey densities. However, it is worth noting that these competitive outcomes are most definitive at extreme values of prey density; in a broad intermediate region, the theoretical outcome is blurred by elements of the dynamics, such as turbulence replenishing prey locally in an area where active zooplankton were just beginning to grow and assert their dominance. Although studying such dynamics is beyond the scope of this section (where we simply want to establish the qualitative existence of the two behavioral regimes), they are interesting avenues for future work, especially work that tries to study the specific details of a diverse zooplankton community.

The framework may require other important modifications to maximize its practical application to real-world cases, since here we assumed simplified predator-prey behavior, hydrodynamics, and environmental context. Marine flows, for example, are three-dimensional and thus vertical velocities also need to be taken into account [64–66], leading to more complicated spatial structures and spatio-temporal correlations of the patterns. The combination of vertical and lateral mixing can generate sharp biological gradients along the edges of the pattern that cannot be captured by our simplistic upwelling events. Thus, future work is needed to understand how vertical gradients and zooplankton behavior interact to shape the profile of phytoplankton blooms. Future work might also consider that, in addition to the most limiting resource, there are other environmental factors that can influence phytoplankton growth in space and time, such as temperature or light [67] and that, beyond our single phytoplankton and our two zooplankton behavioral types, in nature these organisms are hugely diverse [68,69].

Including the above may reveal a more nuanced and diverse behavioral landscape than can be obtained from the simplified predator-prey model introduced here as a proof of concept. Relaxing other simplifications, however, did not alter our conclusions. For example, although we did not explicitly consider top-down sources of regulation for zooplankton, such as fish or viruses [70,71], we performed a sensitivity analysis using a quadratic zooplankton mortality term (which is the standard model closure to represent density-dependent enhanced mortality from higher trophic levels) and recovered the same qualitative results (Fig K). That was also the case when we relaxed the assumption of negligible handling/digestion time for zooplankton replacing behavior with a type-II interaction term, or when trying other values for key parameters such as the catching range.

Thus, although the reality of oceanic patterns is much more complex than our models, our results overall support the hypothesis that zooplankton behavior might contribute to shaping prey spatial patterns.

### 2.4 Conclusions

Our multiscale framework provides a previously computationally inaccessible bridge across scales, from the micro- and millimeter-scale of individuals to the centimeter- and meter-scale of species interactions, and ultimately to the kilometer-scale of macropatterns. Built on a mechanistic individual-based model and scaled via a rigorous coarse-graining procedure, our framework is tractable without having to sacrifice crucial biological detail. This allowed us to understand how prey spatial patterns at different scales may be regulated by zooplankton behavioral traits.

At the microscale, we found that patchiness is characterized by two emergent spatial features: an increase in interspecific encounters and an aggregation of zooplankton, both of which are regulated by turbulence, predator swimming speed, and prey density. These findings bring mechanistic insight to existing empirical work. Our framework provides theoretical support for the field and experimental studies on the impact of turbulence on zooplankton feeding behavior and capture success, which have shown that encounters with prey are affected positively by active swimming and negatively by turbulence [30,72,73]. Additionally, previous work observed that reproducing the different capture success at low densities of passive and active feeding behavior requires the use of qualitatively different feeding response functions [61,74]; in our framework, such functions emerged from the analysis of our IBM, thus allowing us to trace mechanistically their origin. Further, our results suggest that the small-scale zooplankton aggregation and its negative dependence on turbulence observed via towed video microscopy [75] can be explained simply from emergent, indirect correlations between zooplankton individuals in active search and feeding of prey; previous models assumed ballistic or random zooplankton swimming behavior, and thus could not reproduce this pattern, or they did so only after invoking attractive social forces between zooplankton individuals (e.g., [31]).

Analyzing simulation data statistically to infer population-level expressions enabled a rigorous mapping that connected scales. Thus, our coarse-graining method allowed us to obtain a density field description that was fully parameterized by species traits and the physical environment. Because, according to Renormalization Theory, “irrelevant” details are lost during coarsening, these equations represent the fundamental mechanisms underlying the interactions between zooplankton and their prey, with density-dependent feeding playing a particularly relevant role in the emergent large-scale pattern. Previous attempts to connect individual and population levels either estimated population-level encounter rates by, for example, resorting to heuristic, geometry-based, arguments [30]; calculated feeding flow using hydrodynamical models [36,76]; or developed analytical approximations to account for attraction-repulsion between individuals [31]. However, in those approaches the effects of collective emergent phenomena (e.g., density-dependent encounters) were not accounted for, mostly due to the intrinsic complications when dealing with spatially-extended systems [77,78]. Therefore, existing models could not provide a mechanistic link between individual and population levels.

Overcoming this theoretical “problem of scale” allowed us to access valuable ecological information about plankton communities across scales. In particular, we were able to map the curvature of macroscale prey patterns to the swimming speed of the grazing zooplankton, which revealed spatial changes in dominant zooplankton behavior driven by the relative costs and benefits of passive versus active feeding. Our approach thus provides what, to our knowledge, is the first bottom-up framework to identify predator behavior from macroscale prey patterns. Establishing a two-way connection across scales could, for example, be used to monitor which zooplankton (or, at least, which feeding behavior) dominate during the different stages of an upwelling event at given spatial locations. This information can be helpful for understanding not only the dynamics of the microbial loop and oceanic biogeochemistry, but also any associated ecosystem services (e.g., fisheries with preference for particular zooplankton types). Note that, although nutrients and other sources of regulation, such as viruses, are also expected to affect the shape of oceanic macroscopic patterns such as those associated with upwelling events, the robustness of our results against, e.g., different nutrient spatial profiles emphasizes the importance of feeding behavior as a key driving factor for the curvature of the pattern.

We focused here on the perception and active swimming of a generic zooplankton to demonstrate the power of a framework that accounts for individual-level detail; in the future, extensions of our framework could easily be made to incorporate more specific aspects of feeding that have been shown to be important for pattern formation at small scales, such as social interactions [31] and gyrotaxis [27]. A further developed version of our framework that captures how behavior varies across taxa could explain mechanistically patterns of structural variability that have been recently cataloged [12]. Because the expectation is that changes in environmental conditions will alter feeding behavior [61], our framework provides a diagnostic (if not predictive) tool to identify those behavioral changes through the analysis of macroscale patterns. For example, climatic changes affecting zooplankton physiologically and/or the strength of turbulence will alter the costs and benefits of active feeding: if, for instance, short- or long-term climatic changes increase turbulence, higher mixing will make the investment in active feeding less beneficial for zooplankton, and therefore curvature will transition from non-zero to zero slope at smaller *p* values. This means that sharp edges will be found only in more prey-depleted areas of a pattern, and therefore overall patterns will look more generically smooth. Nonetheless, although our methodology suggests a non-invasive way to make inferences regarding prey and zooplankton interactions and behavior (that is, by measuring the sharpness of remote-sensing images of prey macroscopic patterns), the validation of such inferences will still require a combination of such remote-sensing images with species-specific parametrization of behavioral and physiological responses to environmental conditions [61,79].

Beyond the marine context, our computational multiscale framework can be easily applied to and be useful when analytical and computational approaches to scale up individual-level behavior are challenging: for example, when organisms are moving based on complex decision-making strategies [80] that account for the different costs and benefits of individual actions (e.g., fish schools and starling flocks aiming to avoid predatory attacks) [81]. For such cases, coupling with our coarsening method either a mechanistic IBM tailored to the focal system or empirical individual-level data enables an exact and mechanistic connection between scales. This approach provides an alternative to the strong mathematical barriers that arise in analytical methods [31,77] with which to check for potential behavioral signatures in large-scale patterns.

## 3 Materials and methods

### 3.1 Individual-based model simulations

Individuals exist within a square domain of size *L* with periodic boundary conditions (i.e., torus topology). The individual-based model (IBM) accounts for prey and zooplankton birth-death processes, zooplankton feeding behavior, and the influence of an external flow (Sect 2.1). We implemented these processes using a time-dependent Gillespie algorithm [82] for the birth-death events, in combination with a continuous advection process (see S1 *Supplementary materials* for detailed description).

We parameterized our model using values that are generically applicable to zooplankton and their prey (see Table A in S1 *Supplementary materials*). For the former, we aimed to capture the range of behaviors observed from microzooplankton to fish larvae, which is therefore also reflected in the choice of parameter values for the predator and the prey. Thus, we set the prey reproduction rate $r=1\;{\text{day}}^{-1}$ and the zooplankton mortality rate $m=0.1\;{\text{day}}^{-1}$. For computational convenience, we set the prey carrying capacity at *K* = 100 individuals/ml. Zooplankton catching range, $R_{c}$, and perceptual range, $R_{p}$, were set as a function of body size (i.e., the effective radius, set to $B_{g}=0.2\;\text{cm}$ for our generic zooplankton [30]) as $R_{c}=B_{g}$, $R_{p}=5B_{g}$. For the chosen body size, zooplankton velocities can reach values around $V_{z}\sim 0.5$ cm/s [30], which will be our ‘focal’ feeding speed for active zooplankton.

With this parameterization and considering a search rate $c=30\;ind^{-1}{\text{day}}^{-1}$, ingestion rates ranged from a couple of prey per minute to a prey individual every few seconds depending on prey availability, which is within observations (see experimental reference values in, e.g., [83,84]). For more details see Fig F. Other parameterizations did not alter qualitatively our conclusions (see Table A in S1 *Supplementary materials*).

### 3.2 Spatial characterization of the microscale patchiness

To quantify the interspecific correlations of the emergent patterns, we monitored through time the number of prey that were accessible to each zooplankton individual (i.e., within its catching range, $R_{c}$). For a fixed pair of prey and predator densities, (*p*, *z*), the per-predator average number of potential targets reached a constant value $\langle P|R_{c}\rangle$ after a short transient (Fig HA). Analogously, to quantify predator aggregation we measured the stationary value of the number of zooplankton individuals that were within a focal predator’s catching range, $\langle Z|R_{c}\rangle$ (Fig HB). Using these two measures, we calculated the encounter index *E*, which quantifies how much prey availability there is per predator, and the clumping index *C*, which quantifies predator aggregation, both relative to the values expected for the well-mixed scenario [31]:


$$\begin{array}{c} E=\frac{\langle P|R_{c}\rangle }{\pi R_{c}^{2}p}-1\;\text{and}\;C=\frac{\langle Z|R_{c}\rangle }{\pi R_{c}^{2}(z-1/L^{2})}-1\;. \end{array}$$ (6)

where the denominators represent the expected prey targets and zooplankton individuals, respectively, for the well-mixed case. With the definitions above, *E* = *C* = 0 when the community is well-mixed (i.e., individuals are randomly distributed in space); a positive spatial correlation between prey and zooplankton is indicated by *E* > 0; and correlation among zooplankton, by *C* > 0.

### 3.3 Point-vortex model

In order to account for the key elements of turbulence at large-scales, we introduced a fixed number of vortices (or eddies) $N_{e}=1000$ in our spatial domain. Setting the variability of the size and rotation direction of the eddies in a realistic way leads to a stirring process with scale-free velocity spectrum [34,85]. We used the following stream function to capture these features:


$$\begin{array}{c} \Psi (\vec{r},t)=\psi \sum_{n=-\infty }^{+\infty }\sum_{i}^{N_{e}}\omega_{i}(R_{i}^{e}{)}^{2}e^{-|\vec{r}-{\vec{r}}_{i}+nL{|}^{2}/R_{i}^{2}}\;, \end{array}$$ (7)

where ψ controls the angular speed of eddies (in units of day^−1^), $\omega_{i}\in \{-1,1\}$ sets the rotation direction, while $R_{i}^{e}$ and ${\vec{r}}_{i}$ are the radius and center of each eddy *i* (for $i=1,2,\ldots ,N_{e}$). Due to the imposed periodic boundary conditions, the influence of the vortices looped around the domain, which was accounted for by the sum over *n*; since this effect decays exponentially fast, however, considering only n=−2,−1,…,2 already achieved a precision $𝒪(10^{-30})$. The flow velocity, $v_{f}$, is the curl of the stream function:


$$\begin{array}{cc} {\vec{v}}_{f}(\vec{r},t) & =\nabla \times \Psi \;,\\ & =(-\partial \Psi (\vec{r},t)/\partial y,\partial \Psi (\vec{r},t)/\partial x)\;. \end{array}$$ (8)

We sampled vortex sizes from a power-law probability distribution, $P(R^{e})=(R^{e}{)}^{-3}$ (for $R^{e}\in [ℒ/40,ℒ/4]$) and obtained a scale-free transversal velocity spectrum $\propto k^{-3}$, where *k* is the spatial frequency (Fig E). This probability distribution generated a power-law spectrum with exponent −3, characteristic of geostrophic turbulence occurring at large scales [34].

### 3.4 Adaptive-feeding model

The adaptive-feeding model modifies Eq 3 to account for a metabolically mediated switch between passive and active behavior. Specifically, the model introduces two modifications that aim to reflect the costs and benefits of each of the two feeding strategies.

The first change consists in replacing the feeding function, *g*(*p*), by:


$$\begin{array}{c} \tilde{g}(p)=\left\{ \begin{array}{cc} g_{0}=c_{0}c\pi R_{c}^{2}p, & \text{if }\Delta f(p)<0\\ g_{1}=c\pi R_{c}^{2}p(1+E(p)), & \text{if }\Delta f(p)\geq 0 \end{array}\right., \end{array}$$ (9)

where *c*_0_ > 1 reflects the reduced energy expenditure and reduced risk of feeding-related predation associated with passive feeding [63,86]. To assess the sensitivity of our findings to this new parameter, we explored a broad range for $c_{0}\in [2,100]$, which can result from a variety of factors; for example, passive zooplankton are 2–8 times less likely to be predated upon than active zooplankton [87], which motivated the moderate value *c*_0_ = 10 chosen for illustration purposes in Fig 4A [88].

The second change consists in modifying the mortality rate *m* for active predators which, since the term reduces growth, can be seen also as a proxy for metabolic costs:


$$\begin{array}{c} \tilde{m}(p)=\left\{ \begin{array}{cc} m_{0}, & \text{if }\Delta f(p)<0\\ m_{1}=m_{0}qV_{z}^{2}, & \text{if }\Delta f(p)\geq 0 \end{array}\right., \end{array}$$ (10)

where *q* is a fixed hydromechanical factor and $qV_{z}^{2}$ reflects the energy dissipated due to drag, consistent with Stokes’ Law [63].

The behavioral switch is controlled by the relative difference in associated “fitness” $\Delta f(p)=(f_{1}-f_{0})/f_{0}$, where $f_{0}=g_{0}/m_{0}$ and $f_{1}=g_{1}/m_{1}$ are simply defined as the ratio between the benefits and costs of the corresponding behavior [63].

Altogether, these modifications extend our previous model by introducing a trade-off between the two behaviors: the energy-saving but potentially unproductive passive behavior versus the expensive but targeted active behavior.

### 3.5 Measuring the curvature-density relationship

For simulations, we selected hourly snapshots following the peak of prey density during the upwelling event. Immediately after the peak is when the widest range of prey densities are observed, and feeding starts significantly shaping the (very abundant) prey community. For these reasons, and although the curvature curves looked approximately as power laws at any time along the duration of the upwelling event, our analysis of the curvature-density relation (Eq 5) focused on averages obtained within 4 days following the peak.

For satellite images, we first transformed chlorophyll-a observations into phytoplankton density, assuming a ratio [Chl-a mg/m^3^]:[ind./ml]=1.25, which in turn assumes averaged values for carbon content ($\approx 24\cdot 10^{-6}$ mg per cell) [60] and a chlorophyll-to-carbon conversion factor (≈30 gChla/gC) [59] across phytoplankton species. This transformation establishes a common quantitative basis to compare the curvature–density relationships obtained from modeled population density and chlorophyll-a concentration, without affecting the qualitative features relevant for behavioral inference. Then, we obtained the distances between neighboring pixels by means of a geodesic under the WGS84 ellipsoid, which is necessary to calculate the spatial derivatives involved in the curvature expression (Eq 5). These spatial derivatives were calculated using a linear interpolation of the non-uniform grid. To ensure a wide range of phytoplankton densities, we selected snapshots provided by satellite sensors [7] at locations across the globe where chlorophyll-a activity was consistent with a bloom, favoring observations clear of clouds. Specifically, we selected locations where a spring-summer or fall-winter bloom peak was (or had recently) occurred, which typically led to Chlorophyll-a readings ranging from the low to the high extremes of the detection limit.

For each snapshot (either from simulations or real blooms), we obtained the associated local curvature and density for every pixel; we used a log (base 10) scale, $\left[\log|\nabla^{2}p|,\log p\right]$, to better elucidate a potential linear (or non-linear) relationship. We then binned the data according to prey density, and obtained the expected value of the logarithm of the curvature within each bin, generating the curve $\langle \log|\nabla^{2}p|\rangle$ vs. *p* (Fig PC).

For smooth patterns, the profile becomes flat as prey density goes to zero at the borders, and thus the curvature 𝒞 vanishes; for example, if we had an ideal circular pattern and the boundary of the pattern were described by the smooth function $p=e^{-r}$, where *r* represents the distance to the center of the pattern, the associated curvature would be $𝒞(p)=|\nabla^{2}p|\sim p$, which vanishes as p→0, exhibiting zero sharpness, 𝒮=0. For sharp boundaries, in contrast, the density profile drops abruptly to zero, and concurrently so does the curvature to a non-zero value; as an example, a sharp radial density profile given by $p(r)=1-r^{2}$ if *r* < 1 and *p* = 0 if *r* > 1 leads to a constant curvature $𝒞=|\nabla^{2}p|=2>0$ yielding 𝒮=1 even at the pattern boundary, where p→0.

## Supporting information

S1Supplementary materials.**Fig A: Fraction of time with prey in reach.** Solid lines show the fraction of time between ingestion events for which zooplankton have prey in reach but do not capture/ingest them. For high prey densities, as expected, prey individuals are within reach at all times regardless of feeding behavior. For low prey densities (regime for which feeding behavior matters most and the time between events is longer, see main text) active grazers with the typical speed of an active-like predator, $V_{z}=0.5$ cm/s, show a fraction of time spent that is under 0.1; the fraction is ≃0 for the passive case. Fraction of time obtained from data (dots), estimated using the statistics of the number of prey within reach $n_{p}$ (see Fig M), under the assumption that $n_{p}$ follows a Poisson statistics with mean *E* given by Eq 1 in main text. Specifically, the characteristic time-window size within which $n_{p}>0$ is $1/(1-q_{0})$, and with $n_{p}=0$ is 1/*q*_0_, where *q*_0_ is the probability of having $n_{p}=0$ prey in reach in a given instant; thus, the fraction of time for which $n_{p}>0$ is then $q_{0}/(1-q_{0})$ (solid lines). For these results, $\sigma_{\psi }=0.25$ cm/s; remaining parameters as in the main text. **Fig B: Encounter dynamics in the toy model. Left:** encounter index, *E*, as a function of swimming speed. Solid lines represent the encounter index as a function of $V_{z}$ under low small-scale turbulence ($\sigma_{\psi }=0.01$). The different lines correspond to different catching ranges $R_{c}=0.1$ cm (dahsed line), 0.2 cm (solid line), 0.4 cm (dotted line) while the distance between prey was fixed at *d* = 0.15. In general, for $R_{c}\in [d,2d]$ (solid line), prey availability depends non-monotonically on $V_{z}$. Therefore, whenever the presence of this configuration is abundant, e.g., low turbulence, a peak in the curve $E(V_{z})$ will appear. **Right:** probability distribution for the predator position in the toy model for swimming speeds above and below the optimal one ($V_{z}\approx 1$). At optimal swimming speed, the predator confines itself in between the two prey, which maximizes the encounter index. Below optimal swimming speed the predator can be found far from the prey individuals, and above the optimal swimming speed the predator is focused only on one of the prey, leading in both cases to values of *E* that are lower than that corresponding to the optimal speed. **Fig C: Changes in particle number.** Panels show the average offspring number for prey (left) and the average number of zooplankton deaths (right) during Δt=1h. Pixel size *L* = 20 cm; other relevant parameter values are listed on Table A. This particular simulation was done assuming moderate small-scale turbulence, $\sigma_{\psi }=0.25$ cm/s, and swimming speed $V_{z}=0.5$ cm/s, but results qualitatively similar for other values. **Fig D: Flux between pixels.** Coefficients of the cross-pixel-swimming rate as a function of zooplankton swimming speed. For these plots, we used a 4-by-4 lattice (ℒ=40) with pixel size *L* = 10 cm subjected to turbulence intensity $\sigma_{\psi }=0.25$ cm/s (eddy structures were neglected for simplicity), fixing densities (*p*,*z*)=(5,1). See Fig M for scaling and probability distribution. **Fig E: Turbulence model.** In our multiscale framework, we used two models to describe the flow velocity field at small and large scales. At large scales, we used a point-vortex model that accounts for many eddies with different size and rotation direction to generate the realistic features of turbulence. In panel A) the vector field show the velocities induced by a single eddy. Colors indicate the intensity of the speed, which reaches a maximum for intermediate distance from the eddy center. For spatial scales below the pixel size, we used a simplified flow model where the velocities are random vectors (see *Materials and methods*), as shown in panel B. The combination of both models allow for the continuity of the flow velocity spectrum as shown in panel C). For large scales, the velocity field is structured and characterized by a power-law scaling (see blue line produced using the point-vortex model) that is typically observed in the ocean [34]. At small scales, any coherent structure is expected to be fade leaving tiny velocity fluctuations, which are preserved by the random flow model (gray solid line). Vertical dashed lines highlight eddy maximum and minimum sizes, *R*_max_ and *R*_min_ respectively, and *L*, the pixel size (which is 1 in the scaled horizontal axis in panel C). The above results use turbulence intensity ψ=0.6, leading to an average speed of 1km/day in a 120m square domain and $\sigma_{\psi }=0.25$ cm/s. **Fig F: Timescale separation analysis.** Red and purple dots show the values of the feeding and spatial timescales, in seconds, for $V_{z}=0.1$ (left panel), $V_{z}=0.5$ (middle panel), and $V_{z}=1.0$ cm/s (right panel). Feeding timescale is defined as the average inter-ingestion time, and spatial timescale is defined as the characteristic relaxation times of the spatial index *E* (see Fig H). We can conservatively define the condition for timescale separation as spatial relaxation being 10 times faster than feeding, i.e., when feeding dots are inside the white region in the figure. In this regime, which occurs approximately for $p\in [10^{-3},10^{4}]$ in our $V_{z}=0.5$ reference case, we can approximate the encounter index as E(t)≃E(p). Results shown are for small-scale turbulence used in the manuscript figures, $\sigma_{\psi }=0.25$ cm/s. Lines show the theoretical expectations for the behavior of the timescales as a function of prey density: red solid line (feeding timescale) is directly given by 1/*g*(*p*) while the purple dashed line represent the scaling relation, $1/p^{\gamma }$, where we selected γ=0.5 for $V_{z}=0.1$ and γ=1.0 for $V_{z}=0.5$ and 1.0, to guide the trend and provide a visual boundary to the region with timescale separation. System size was chosen within the range from *L* = 20 to *L* = 500 cm to ensure the presence of at least a few hundreds of prey individuals. As shown in Fig A, this timescale separation is *not* an artifact of the simulation algorithm, that is, it does not result from an unrealistic “waiting without eating” period between demographic events. Note that, for the passive zooplankton case $V_{z}=0$, there is no spatial structure and therefore the spatial timescale is zero. **Fig G: Pair-correlation functions.** Pair-correlation function (PCF) between prey (PP), zooplankton (ZZ), and between zooplankton and prey (ZP) for the case shown in Fig 2A. where $\left(\sigma_{\psi },V_{z})=(0.25,0.5\right)$ and *L* = 20 cm (see Fig 2A caption for more details). For both ZZ and ZP, short scales lead to a substantial positive deviation from the well-mixed scenario (PCF = 1) that indicates: i) higher interspecific encounter rate and ii) zooplankton aggregation. The spatial correlation given by the PP curve, on the other hand, indicates that prey are fairly well-mixed (PCF ≃1). **Fig H: Encounter and clumping index transient and stationary values.** Panels show the transient (A-B) and stationary values (C-F) for the encounter index *E*(*t*) (left column) and clumping index *C*(*t*) (right column), for different swimming speeds and small-scale turbulence intensity, $\sigma_{\psi }$. The first row (panels A and B) shows these indices as a function of time for $\left(\sigma_{\psi },V_{z})=(0.25,0.50\right)$, with stationary values *E* and *C* (highlighted with a solid black line) achieved at characteristic time scales 0.8 and 1.8 seconds, respectively. Second row summarizes the dependence of *E* (panel C) and *C* (panel D) on swimming speed, $V_{z}$, for a given small-scale turbulence intensity, $\sigma_{\psi }$; every curve corresponds to a vertical transect of the heatmaps from the bottom row panels. These heatmaps (panels E and F) illustrate how the tug-of-war between behavior and environment ($V_{z}$ and $\sigma_{\psi }$) affects *E* and *C* and leads to the emergence of correlations among individuals. The specific case $\left(\sigma_{\psi },V_{z})=(0.25,0.50\right)$ shown in A-B is indicated with an arrow in other panels. Simulations were performed starting from the well-mixed state (*E* = *C* = 0) with fixed densities, *p* = 1.28 and *z* = 1, as in Fig 2A-2B (see Fig 2 caption for remaining parameter values). **Fig I: Encounter and Clumping index as a function of predator and prey densities.** Panels A-B complement Fig 2C by showing both spatial indices as a function of prey density for different zooplankton densities, for a fixed swimming speed $V_{z}=0.5$ cm/s. Results for both indices remain robust against changes in zooplankton density *z*, leading to the conclusion that only the dependence on *p* is of relevance. Moreover, the solid line in panel A shows that the power-law of Eq 1 in main text is valid regardless of zooplankton density *z*. See Fig 2 caption for details. **Fig J: Dependence of different aspects of the emergent grazing rate function** *g*(*p***) on** $V_{z}$ **and** $\sigma_{\psi }$**.** A) Parameter $\alpha (\sigma_{\psi },V_{z})=\alpha_{1}(\sigma_{\psi })(1-\exp[-V_{z}/\alpha_{2}(\sigma_{\psi })])$ as a function of swimming speed $V_{z}$ for different turbulence intensities $\sigma_{\psi }$ (color bar), with $\alpha_{1}(\sigma_{\psi })=10.61\exp(-2.32\sigma_{\psi })$ and $\alpha_{2}(\sigma_{\psi })=6.99\sigma_{\psi }^{1.66}$. B) Exponent β as a function of swimming speed $V_{z}$, for different turbulence intensities $\sigma_{\psi }$ (color bar). Each α and β value was obtained via minimization of the sum of squared residuals (i.e., least-squares estimation) of a power-law function linking encounter index and phytoplankton density in log-log space; the latter linearizes the relationship, with slope corresponding to −β and intercept determining α. Repeating the fitting exercise for different swimming speeds and turbulence strength values provided the panels A and B data. C-D) Per-capita grazing rate for low C) and reference D) swimming speeds under different turbulence intensities (same parametrization as panels A and B). **Fig K: Dependence of results on catching range and zooplankton mortality.** Left: Encounter index as a function of phytoplankton concentration for different values of catching range, $R_{c}\in \{0.2,0.3,0.4,0.5,1.0\}$, for fixed perceptual range, $R_{p}$=1.0, turbulence intensity $\sigma_{\psi }=0.25$ and swimming speed $V_{z}=0.5$. Right: Relationship between curvature and phytoplankton concentration for a single realization of a simulated bloom under the conditions of Fig 3. “Linear” refers to our current model, where zooplankton mortality is a linear function of zooplankton density; “quadratic” refers to results produced by a modified version of the model where zooplankton mortality is represented by a quadratic function of zooplankton density. **Fig L: Spatial indices become invariant beyond** L≃10 **cm.** Encounter and clumping indices as a function of pixel size *L* (dots). Beyond L≃10, the standard deviations (error bars) vanish and the values of the spatial indices achieve the convergence values, indicated by thick solid lines. For this plot, (*p*,*z*)=(1,5) and $\left(\sigma_{\psi },V_{z})=(0.25,0.5\right)$, representing one of the cases in Fig 2B (where *L* = 20 was used); see Fig 2 caption for rest of simulation parameters. **Fig M: Coarse-scale statistics.** A-B) Normalized histogram for the number of prey ingested during 1 hour, under different (fixed) prey densities, within a pixel size *L* = 20 cm (panel A); and for the number of cross-pixel-swimming events due to small-scale turbulence and swimming (panel B). C-D) Size-scaling for the coefficient of variation for ingestion (panel C) and the mean number of cross-pixel-swimming events (panel D) for *p* = 5 ind/cm^2^. E-F) Normalized histogram for the number of prey ingested during 1 hour (panel E) and for the number of cross-pixel-swimming events due to swimming (panel F) for $V_{z}=0.0$ cm/s (black) and $V_{z}=0.5$ cm/s (purple) and homogeneous environments (thus ∇p≈0). G) Mean 1-hour ingestion for the case of panel E) as a function of prey density, *p*, for different swimming speeds, $V_{z}$. Crosses mark the case represented in panel E. H) Mean number cross-pixel-swimming events as a function of “chemotactic signal”, ∇p/p for different swimming speeds. Crosses mark the ∇p/p≈0 case of panel F. Solid and dashed lines in panels A, B, E, F are Gaussian fits assuming equal mean and variance (an approximation for the Poisson distribution). Small-scale turbulence intensity is $\sigma_{\psi }=0.25$ cm/s, search rate *c* = 1.0 day^−1^, *L* = 20 cm, and species density (*p*,*z*)=(5,1), unless otherwise specified. See *Supplementary materials*  for details. **Fig N: Active behavior leads to unstable community dynamics.** Trajectories for the passive ($V_{z}=0.0$, panel A) and active zooplankton ($V_{z}=0.5$ cm/s, panels B and C) cases with low search rate *c* = 2 day^−1^ (panels A and B) and high search rate, *c* = 5 day^−1^ (panel C). Trajectories were obtained numerically integrating, $\dot{p}=F_{p}(p,z)$ and $\dot{z}=F_{z}(p,z)$ (Eqs 1 and 2 without the noises), with different initial conditions. Time increases from white to black color, with arrows highlighting the trajectories that lead to coexistence (blue) and extinction of one of the species (red). Results show that, as active behavior becomes more relevant (i.e., from panel A to C), the community dynamics become more unstable and thereby prone to extinction. This conclusion is supported by the linear stability analysis shown in panels D-E as follows. D) The system has only one (non-trivial) homogeneous state $\left(p^{⋆},z^{⋆}\right)$, shown as a function of the predator search rate, *c*. E) Performing a small perturbation around the homogeneous state, we obtained the real and imaginary parts of the dominant eigenvalue (eigenvalue with the largest real part); positive and negative real part suggest outward and inward spirals, respectively, with the value of the imaginary part corresponding to the angular velocity around $\left(p^{⋆},z^{⋆}\right)$. This linear stability analysis allowed us to predict the qualitative dynamics shown in panels A, B, and C. **Fig O: Different ways to simulate upwelling events.** Carrying-capacity profiles, *K*(*r*,*t*) for the band (top row) and randomly distributed hotpots of nutrient input (bottom row) scenarios at *t* = 0 (left column) and at *t* = 3 days (right column). Colors indicate the carrying capacity value. The steps to simulate the nutrient band profile are described in the main text; for the random hotspots, we simulated the input of nutrient at random locations by using a Gaussian profile for the carrying capacity (i.e., carrying capacity decreasing, from a maximum of 10^4^, following a Gaussian exponential function of the distance to the hotspot center with variance $\sigma^{2}=15$). **Fig P: Dynamics of sharpness.** A) Spatial average of prey density (⟨p⟩, green) and zooplankton density (⟨z⟩, red) as a function of time (left panel) and corresponding trajectory in the (*p*,*z*)-space (right panel). In both panels, solid lines represent results for $V_{z}=0.0$ and dashed lines results for $V_{z}=0.5$ cm/s. Initial condition was p(x,y,t=0)=1≪K=100 and *z*(*x*,*y*,*t* = 0)=0.01 ind/cm^2^. B) Prey density at *t* = 12d (as marked in a) for $V_{z}=0.5$. Color indicates density, in ind/mL, in log-scale to highlight the differences when predators are active. See Fig 3 caption for simulation details. C) Curvature as a function of prey density for $V_{z}=0$ and $V_{z}=0.5$ for three snapshots at *t* = 2, *t* = 12 and *t* = 14 d (from lef*t* to right). The three snapshots show a wide range of densities that led to a clear power-law scaling, still observed at *t* = 14 d. For very long times, for which the upwelling event is in a fading phase, deviations from a power law are expected. Snapshot *t* = 12 corresponds to the peak of prey density during the upwelling event, for which prey density spatial distribution is shown in log-scale in Fig 3. D) Curvature-densi*t*y rela*t*ion considering a passive preda*t*or, $V_{z}=0.0$, obtained for different values of the search rate *c* (colors) and different days around the peak of the upwelling event within the observation window (differen*t* lines, t=12−14 d). E) Prey pattern sharpness as a function of time for simulation data shown in panel C, wi*t*h error bars representing standard deviation for sharpness when calculating it from curvature data. **Fig Q: Curvature-density relation sensitivity analysis.** A) Curvature-density relation for different values of the passive-zooplankton parameter *c*_0_, used in the adaptive-feeding model. The vertical dashed lines show the density threshold below which feeding actively is more beneficial than feeding passively, which these data show decreases as *c*_0_ increases. B) Curvature-density relation for two upwelling scenarios: i) band (in blue), for which nutrient is introduced along a centered horizontal line as in Fig 4A, see Fig OA) and ii) hotspots (in gray), for which nutrient is introduced as randomly distributed circular sources (Fig OC). **Fig R: Spatial niche partitioning (2-species model).** A) Curvature-density relationship extracted from the 2-species model. Solid black lines (𝒮=0 and 𝒮=1) indicate the slope, associated with dominance of a particular feeding behavior. B) Prey density and C) dominant-zooplankton-behavior patterns for the 2-species model with $V_{z}=0.5$ cm/s, *q* = 4, $m_{1}=m_{0}=0.10$ day^−1^, *c*_0_ = 10 (chosen using ecological arguments, see *Materials and methods* for details). Remaining parameters are the same as in Fig 3A. Dominance is defined as the relative difference between active and passive zooplankton densities, $100\cdot (z_{1}-z_{0})/z_{0}$. Thus, + 50% dominance implies 50% more abundant active predators than passive at that specific time and location. **Fig S: Data summary. A)** Locations for analyzed blooms across the globe, with seasons indicated by up (spring-summer) and down (fall-winter) triangles (see Table C) provided by the Sea-Hawk initiative [7] at 120m pixel-size resolution. **B)** Curvature-density relationship for the real blooms in panel **A.** Crosses represent data for all snapshots, while bloom #4 (highlighted in panel A and shown in Fig 1B) is indicated with filled circles. Squares show, for comparison, the curvature-density relationship using MODIS-Aqua data (1km pixel-size resolution) for same location and time of bloom #4. Shades of green indicate the number of pixels used for each phytoplankton-density bin (pixel count, see color bar). Red and blue lines indicate the slope expected for passive and active behavior.(PDF)

## References

[1] Falkowski P. Ocean science: the power of plankton. Nature. 2012;483(7387):S17-20. doi: 10.1038/483S17a 22378122

[2] Kiørboe T. Formation and fate of marine snow: small-scale processes with large- scale implications. Sci Mar. 2001;65(S2):57–71. doi: 10.3989/scimar.2001.65s257

[3] Steinberg DK, Landry MR. Zooplankton and the ocean carbon cycle. Ann Rev Mar Sci. 2017;9:413–44. doi: 10.1146/annurev-marine-010814-015924 27814033

[4] Kiørboe T, Jackson GA. Marine snow, organic solute plumes, and optimal chemosensory behavior of bacteria. Limnology & Oceanography. 2001;46(6):1309–18. doi: 10.4319/lo.2001.46.6.1309

[5] Barbier EB. Marine ecosystem services. Curr Biol. 2017;27(11):R507–10. doi: 10.1016/j.cub.2017.03.020 28586688

[6] Rohr T, Richardson AJ, Lenton A, Chamberlain MA, Shadwick EH. Zooplankton grazing is the largest source of uncertainty for marine carbon cycling in CMIP6 models. Commun Earth Environ. 2023;4(1). doi: 10.1038/s43247-023-00871-w

[7] NASA Goddard Space Flight Center, Ocean Ecology Laboratory, Ocean Biology Processing Group. HawkEye-SeaHawk Ocean Color Data; 2018 Reprocessing. Greenbelt, MD, USA: NASA OB.DAAC; 2022. 10.5067/SEAHAWK/HAWKEYE/L2/OC/2018

[8] NASA Goddard Space Flight Center, Ocean Ecology Laboratory, Ocean Biology Processing Group. Moderate-resolution Imaging Spectroradiometer (MODIS) Aqua Ocean Color Data; 2022 Reprocessing. Greenbelt, MD, USA: NASA OB.DAAC; 2022. 10.5067/AQUA/MODIS/L2/OC/2022

[9] Basedow SL, McKee D, Lefering I, Gislason A, Daase M, Trudnowska E, et al. Remote sensing of zooplankton swarms. Sci Rep. 2019;9(1):686. doi: 10.1038/s41598-018-37129-x 30679810 PMC6346024

[10] Buitenhuis ET, Hashioka T, Quéré CL. Combined constraints on global ocean primary production using observations and models. Global Biogeochemical Cycles. 2013;27(3):847–58. doi: 10.1002/gbc.20074

[11] Laber CP, Hunter JE, Carvalho F, Collins JR, Hunter EJ, Schieler BM, et al. Coccolithovirus facilitation of carbon export in the North Atlantic. Nat Microbiol. 2018;3(5):537–47. doi: 10.1038/s41564-018-0128-4 29531367

[12] Robinson KL, Sponaugle S, Luo JY, Gleiber MR, Cowen RK. Big or small, patchy all: Resolution of marine plankton patch structure at micro- to submesoscales for 36 taxa. Sci Adv. 2021;7(47):eabk2904. doi: 10.1126/sciadv.abk2904 34797707 PMC8604402

[13] Lombard F, Koski M, Kiørboe T. Copepods use chemical trails to find sinking marine snow aggregates. Limnology & Oceanography. 2012;58(1):185–92. doi: 10.4319/lo.2013.58.1.0185

[14] Everett JD, Baird ME, Buchanan P, Bulman C, Davies C, Downie R, et al. Modeling what we sample and sampling what we model: challenges for zooplankton model assessment. Front Mar Sci. 2017;4. doi: 10.3389/fmars.2017.00077

[15] Walters CJ, Holling CS. Large‐scale management experiments and learning by doing. Ecology. 1990;71(6):2060–8. doi: 10.2307/1938620

[16] Jackson G, Kiørboe T. Zooplankton use of chemodetection to find and eat particles. Mar Ecol Prog Ser. 2004;269:153–62. doi: 10.3354/meps269153

[17] Raina J-B, Lambert BS, Parks DH, Rinke C, Siboni N, Bramucci A, et al. Chemotaxis shapes the microscale organization of the ocean’s microbiome. Nature. 2022;605(7908):132–8. doi: 10.1038/s41586-022-04614-3 35444277

[18] Humphries S. Filter feeders and plankton increase particle encounter rates through flow regime control. Proc Natl Acad Sci U S A. 2009;106(19):7882–7. doi: 10.1073/pnas.0809063106 19416879 PMC2683099

[19] Heneghan RF, Everett JD, Blanchard JL, Sykes P, Richardson AJ. Climate-driven zooplankton shifts cause large-scale declines in food quality for fish. Nat Clim Chang. 2023;13(5):470–7. doi: 10.1038/s41558-023-01630-7

[20] Strömberg KHP, Smyth TJ, Allen JI, Pitois S, O’Brien TD. Estimation of global zooplankton biomass from satellite ocean colour. Journal of Marine Systems. 2009;78(1):18–27. doi: 10.1016/j.jmarsys.2009.02.004

[21] Druon J-N, Hélaouët P, Beaugrand G, Fromentin J-M, Palialexis A, Hoepffner N. Satellite-based indicator of zooplankton distribution for global monitoring. Sci Rep. 2019;9(1):4732. doi: 10.1038/s41598-019-41212-2 30894610 PMC6427021

[22] Brewin RJW, Sathyendranath S, Platt T, Bouman H, Ciavatta S, Dall’Olmo G, et al. Sensing the ocean biological carbon pump from space: a review of capabilities, concepts, research gaps and future developments. Earth-Science Reviews. 2021;217:103604. doi: 10.1016/j.earscirev.2021.103604

[23] Anderson PW. More is different: broken symmetry and the nature of the hierarchical structure of science. Science. 1972;177(4047):393–6. 17796623 10.1126/science.177.4047.393

[24] Levin SA, Segel LA. Hypothesis for origin of planktonic patchiness. Nature. 1976;259(5545):659–659. doi: 10.1038/259659a0814470

[25] Vilar JMG, Solé RV, Rubí JM. On the origin of plankton patchiness. Physica A: Statistical Mechanics and its Applications. 2003;317(1–2):239–46. doi: 10.1016/s0378-4371(02)01322-5

[26] Taylor JR, Stocker R. Trade-offs of chemotactic foraging in turbulent water. Science. 2012;338(6107):675–9. doi: 10.1126/science.1219417 23118190

[27] Durham WM, Climent E, Barry M, De Lillo F, Boffetta G, Cencini M, et al. Turbulence drives microscale patches of motile phytoplankton. Nat Commun. 2013;4:2148. doi: 10.1038/ncomms3148 23852011

[28] Brumley DR, Carrara F, Hein AM, Yawata Y, Levin SA, Stocker R. Bacteria push the limits of chemotactic precision to navigate dynamic chemical gradients. Proc Natl Acad Sci U S A. 2019;116(22):10792–7. doi: 10.1073/pnas.1816621116 31097577 PMC6561191

[29] Wheeler JD, Secchi E, Rusconi R, Stocker R. Not just going with the flow: the effects of fluid flow on bacteria and plankton. Annu Rev Cell Dev Biol. 2019;35:213–37. doi: 10.1146/annurev-cellbio-100818-125119 31412210

[30] Kiørboe T. How zooplankton feed: mechanisms, traits and trade-offs. Biol Rev Camb Philos Soc. 2011;86(2):311–39. doi: 10.1111/j.1469-185X.2010.00148.x 20682007

[31] Flierl G, Grünbaum D, Levins S, Olson D. From individuals to aggregations: the interplay between behavior and physics. J Theor Biol. 1999;196(4):397–454. doi: 10.1006/jtbi.1998.0842 10036198

[32] Steele JH, Henderson EW. A simple model for plankton patchiness. J Plankton Res. 1992;14(10):1397–403. doi: 10.1093/plankt/14.10.1397

[33] Powell TM, Okubo A. Turbulence, diffusion and patchiness in the sea. Philosophical Transactions of the Royal Society of London Series B: Biological Sciences. 1994;343(1303):11–8. doi: 10.1098/rstb.1994.0002

[34] Abraham ER. The generation of plankton patchiness by turbulent stirring. Nature. 1998;391(6667):577–80. doi: 10.1038/35361

[35] Serra-Pompei C, Soudijn F, Visser AW, Kiørboe T, Andersen KH. A general size- and trait-based model of plankton communities. Progress in Oceanography. 2020;189:102473. doi: 10.1016/j.pocean.2020.102473

[36] Nielsen LT, Asadzadeh SS, Dölger J, Walther JH, Kiørboe T, Andersen A. Hydrodynamics of microbial filter feeding. Proc Natl Acad Sci U S A. 2017;114(35):9373–8. doi: 10.1073/pnas.1708873114 28808016 PMC5584453

[37] Hanazato T. Pesticide effects on freshwater zooplankton: an ecological perspective. Environ Pollut. 2001;112(1):1–10. doi: 10.1016/s0269-7491(00)00110-x 11202648

[38] Prairie JC, Sutherland KR, Nickols KJ, Kaltenberg AM. Biophysical interactions in the plankton: a cross‐scale review. Limn Fluids and Environments. 2012;2(1):121–45. doi: 10.1215/21573689-1964713

[39] Utne-Palm AC. Visual feeding of fish in a turbid environment: physical and behavioural aspects. Marine and Freshwater Behaviour and Physiology. 2002;35(1–2):111–28. doi: 10.1080/10236240290025644

[40] Lunt J, Smee DL. Turbidity interferes with foraging success of visual but not chemosensory predators. PeerJ. 2015;3:e1212. doi: 10.7717/peerj.1212 26401444 PMC4579029

[41] Gregory B, Christophe L, Martin E. Rapid biogeographical plankton shifts in the North Atlantic Ocean. Global Change Biology. 2009;15(7):1790–803. doi: 10.1111/j.1365-2486.2009.01848.x

[42] Franks P. Plankton patchiness, turbulent transport and spatial spectra. Mar Ecol Prog Ser. 2005;294:295–309. doi: 10.3354/meps294295

[43] Vermeij GJ. Unsuccessful predation and evolution. The American Naturalist. 1982;120(6):701–20. doi: 10.1086/284025

[44] Ryderheim F, Thygesen UH, Kiørboe T. Short handling times allow for active prey selection in suspension feeding copepods. Limnology & Oceanography. 2023;68(4):891–901. doi: 10.1002/lno.12317

[45] Kiørboe T, Visser A. Predator and prey perception in copepods due to hydromechanical signals. Mar Ecol Prog Ser. 1999;179:81–95. doi: 10.3354/meps179081

[46] Morozov AY. Emergence of Holling type III zooplankton functional response: bringing together field evidence and mathematical modelling. J Theor Biol. 2010;265(1):45–54. doi: 10.1016/j.jtbi.2010.04.016 20406647

[47] Rohr T, Richardson AJ, Lenton A, Shadwick E. Recommendations for the formulation of grazing in marine biogeochemical and ecosystem models. Progress in Oceanography. 2022;208:102878. doi: 10.1016/j.pocean.2022.102878

[48] Wilson KG. The renormalization group and critical phenomena. Rev Mod Phys. 1983;55(3):583–600. doi: 10.1103/revmodphys.55.583

[49] Goldenfeld N. Lectures on phase transitions and the renormalization group. CRC Press; 2018.

[50] Hill R. Elastic properties of reinforced solids: some theoretical principles. Journal of the Mechanics and Physics of Solids. 1963;11(5):357–72. doi: 10.1016/0022-5096(63)90036-x

[51] Bird GA. The DSMC method. CreateSpace Independent Publishing Platform; 2013.

[52] Bjørnstad ON, Bascompte J. Synchrony and second‐order spatial correlation in host–parasitoid systems. Journal of Animal Ecology. 2001;70(6):924–33. doi: 10.1046/j.0021-8790.2001.00560.x

[53] Wiegand T, Wang X, Anderson-Teixeira KJ, Bourg NA, Cao M, Ci X, et al. Consequences of spatial patterns for coexistence in species-rich plant communities. Nat Ecol Evol. 2021;5(7):965–73. doi: 10.1038/s41559-021-01440-0 33941904 PMC8257505

[54] Holling CS. The functional response of predators to prey density and its role in mimicry and population regulation. Mem Entomol Soc Can. 1965;97(S45):5–60. doi: 10.4039/entm9745fv

[55] Murray JD. Mathematical biology: I. An introduction. Springer Science & Business Media; 2007.

[56] Boyd PW, Claustre H, Levy M, Siegel DA, Weber T. Multi-faceted particle pumps drive carbon sequestration in the ocean. Nature. 2019;568(7752):327–35. doi: 10.1038/s41586-019-1098-2 30996317

[57] Steele JH. Spatial Pattern in Plankton Communities. Springer Science & Business Media; 1978.

[58] Nayak PR. Random process model of rough surfaces. Journal of Lubrication Technology. 1971;93(3):398–407. doi: 10.1115/1.3451608

[59] Sathyendranath S, Stuart V, Nair A, Oka K, Nakane T, Bouman H, et al. Carbon-to-chlorophyll ratio and growth rate of phytoplankton in the sea. Mar Ecol Prog Ser. 2009;383:73–84. doi: 10.3354/meps07998

[60] Menden-Deuer S, Lessard EJ. Carbon to volume relationships for dinoflagellates, diatoms, and other protist plankton. Limnology & Oceanography. 2000;45(3):569–79. doi: 10.4319/lo.2000.45.3.0569

[61] Kiørboe T, Saiz E, Tiselius P, Andersen KH. Adaptive feeding behavior and functional responses in zooplankton. Limnology & Oceanography. 2017;63(1):308–21. doi: 10.1002/lno.10632

[62] Kiørboe T. Organismal trade-offs and the pace of planktonic life. Biol Rev Camb Philos Soc. 2024;99(6):1992–2002. doi: 10.1111/brv.13108 38855937

[63] Visser AW. Motility of zooplankton: fitness, foraging and predation. Journal of Plankton Research. 2007;29(5):447–61. doi: 10.1093/plankt/fbm029

[64] McWilliams JC. Submesoscale currents in the ocean. Proc Math Phys Eng Sci. 2016;472(2189):20160117. doi: 10.1098/rspa.2016.0117 27279778 PMC4893189

[65] McGillicuddy Jr DJ. Mechanisms of physical-biological-biogeochemical interaction at the oceanic mesoscale. Ann Rev Mar Sci. 2016;8:125–59. doi: 10.1146/annurev-marine-010814-015606 26359818

[66] McGillicuddy Jr DJ, Franks PJS. Models of Plankton patchiness. Encyclopedia of Ocean Sciences. Elsevier. 2019. p. 536–46. 10.1016/b978-0-12-409548-9.11610-0

[67] Litchman E, Klausmeier CA. Trait-based community ecology of plankton. Annu Rev Ecol Evol Syst. 2008;39:615–39.

[68] Follows MJ. Dutkiewicz. Modeling diverse communities of marine microbes. Annual Review of Marine Science. 2011;3:427–51. 10.1146/annurev-marine-120709-142848 21329212

[69] Calbet A, Landry MR. Phytoplankton growth, microzooplankton grazing, and carbon cycling in marine systems. Limnology & Oceanography. 2004;49(1):51–7. doi: 10.4319/lo.2004.49.1.0051

[70] Locke H, Bidle KD, Thamatrakoln K, Johns CT, Bonachela JA, Ferrell BD, et al. Marine viruses and climate change: Virioplankton, the carbon cycle, and our future ocean. Adv Virus Res. 2022;114:67–146. doi: 10.1016/bs.aivir.2022.09.001 39492214

[71] Mitra A. Are closure terms appropriate or necessary descriptors of zooplankton loss in nutrient–phytoplankton–zooplankton type models?. Ecological Modelling. 2009;220(5):611–20. doi: 10.1016/j.ecolmodel.2008.12.008

[72] Kiørboe T, Saiz E. Planktivorous feeding in calm and turbulent environments, with emphasis on copepods. Mar Ecol Prog Ser. 1995;122:135–45. doi: 10.3354/meps122135

[73] Saiz E, Calbet A, Broglio E. Effects of small‐scale turbulence on copepods: The case of Oithona davisae. Limnology & Oceanography. 2003;48(3):1304–11. doi: 10.4319/lo.2003.48.3.1304

[74] Hein AM, McKinley SA. Sensory information and encounter rates of interacting species. PLoS Comput Biol. 2013;9(8):e1003178. doi: 10.1371/journal.pcbi.1003178 23966847 PMC3744405

[75] Davis CS, Gallager SM, Solow AR. Microaggregations of oceanic plankton observed by towed video microscopy. Science. 1992;257(5067):230–2. doi: 10.1126/science.257.5067.230 17794756

[76] Pujara N, Koehl MAR, Variano EA. Rotations and accumulation of ellipsoidal microswimmers in isotropic turbulence. J Fluid Mech. 2018;838:356–68. doi: 10.1017/jfm.2017.912

[77] Barraquand F, Murrell DJ. Scaling up predator–prey dynamics using spatial moment equations. Methods Ecol Evol. 2013;4(3):276–89. doi: 10.1111/2041-210x.12014

[78] Cipriotti PA, Wiegand T, Pütz S, Bartoloni NJ, Paruelo JM. Nonparametric upscaling of stochastic simulation models using transition matrices. Methods Ecol Evol. 2015;7(3):313–22. doi: 10.1111/2041-210x.12464

[79] Perna A, Rivoli ES, Reiss J, Perkins DM. Energy allocation explains how protozoan phenotypic traits change in response to temperature and resource supply. bioRxiv. 2023. 2023–07. 10.1002/ece3.74103 PMC13439638 42559310

[80] Giraldeau LA, Caraco T. Social foraging theory. Social Foraging Theory. Princeton University Press; 2018.

[81] Goldstone RL, Janssen MA. Computational models of collective behavior. Trends Cogn Sci. 2005;9(9):424–30. doi: 10.1016/j.tics.2005.07.009 16085450

[82] Vestergaard CL, Génois M. Temporal Gillespie algorithm: fast simulation of contagion processes on time-varying networks. PLoS Comput Biol. 2015;11(10):e1004579. doi: 10.1371/journal.pcbi.1004579 26517860 PMC4627738

[83] Henriksen C, Saiz E, Calbet A, Hansen B. Feeding activity and swimming patterns of Acartia grani and Oithona davisae nauplii in the presence of motile and non-motile prey. Mar Ecol Prog Ser. 2007;331:119–29. doi: 10.3354/meps331119

[84] Xu J, Nielsen LT, Kiørboe T. Foraging response and acclimation of ambush feeding and feeding‐current feeding copepods to toxic dinoflagellates. Limnology & Oceanography. 2018;63(4):1449–61. doi: 10.1002/lno.10782

[85] Aref H. Point vortex dynamics: a classical mathematics playground. Journal of Mathematical Physics. 2007;48(6). doi: 10.1063/1.2425103

[86] Visser AW, Mariani P, Pigolotti S. Swimming in turbulence: zooplankton fitness in terms of foraging efficiency and predation risk. Journal of Plankton Research. 2008;31(2):121–33. doi: 10.1093/plankt/fbn109

[87] Ohman M. Behavioral responses of zooplankton to predation. Bulletin of Marine Science. 1988;43(3):530–50.

[88] Almeda R, van Someren Gréve H, Kiørboe T. Behavior is a major determinant of predation risk in zooplankton. Ecosphere. 2017;8(2). doi: 10.1002/ecs2.1668

